# 16-Hydroxycleroda-3,13-dien-15,16-olide and *N*-Methyl-Actinodaphine Potentiate Tamoxifen-Induced Cell Death in Breast Cancer

**DOI:** 10.3390/molecules23081966

**Published:** 2018-08-06

**Authors:** Bharath Kumar Velmurugan, Po-Chih Wang, Ching-Feng Weng

**Affiliations:** 1 Faculty of Applied Sciences, Ton Duc Thang University, Ho Chi Minh City, Vietnam; bharath.kumar.velmurugan@tdt.edu.vn; 2 Department of Life Science and Institute of Biotechnology, National Dong Hwa University, Hualien 97401, Taiwan; a2704490@gmail.com

**Keywords:** HCD, MA, TMX, apoptosis, Bcl2, Bax

## Abstract

In this study, we investigated whether 16-hydroxycleroda-3,13-dien-15,16-olide (HCD) and *N*-methyl-actinodaphine (MA) could sensitize breast cancer cells to Tamoxifen (TMX) treatment. MA or HCD alone or in combination with TMX dose-dependently inhibited MCF-7 and MDA-MB-231 cell growth, with a more potent inhibition on MDA-MB 231 cells. Furthermore, this novel combination significantly induced S and G2/M cell cycle phase in MDA-MB 231 than MCF-7 cells. Further determination of the apoptotic induction showed that MA or HCD and TMX combination inhibited MDA-MB-231 and MCF-7 cancer cells by upregulating Bax and by downregulating Bcl-2 mRNA and protein expression without altering Caspase-8 and Caspase-12 expression. These results suggest that MA or HCD pretreatment may potentiate the anti-tumor effect of tamoxifen on breast cancer.

## 1. Introduction

Breast cancer is the most common cancer in women worldwide, and its incidence continues to rise [1]. Breast cancer cells growth is stimulated by estrogen and estrogen receptor (ER) signaling, however this can be blocked by few anti-hormonal drugs, like aromatase inhibitors or the oestrogen receptor antagonists, tamoxifen (TMX) or fulvestrant [2,3]. In particular, tamoxifen has been used in the last 40 years and has resulted in a reduction of the mortality rate by 30% [4]; despite its advantages, acquired resistance remains a significant obstacle to treat estrogen-positive cancers [5]. Previous studies showed that combination therapy with other drugs enhanced tamoxifen activity [6]. Thus, an alternative approach by combining chemotherapeutic agents with phytochemicals that specifically target cancer cells by sensitizing to chemotherapy must be developed [7,8].

16-Hydroxycleroda-3,13-dien-15,16-olide (HCD) is one of the major clerodane diterpenoid compounds isolated from *Polyalthia longifolia* that has shown various anti-inflammatory activities [9,10,11]. HCD has been shown to possess potential cytotoxic effect against leukemia, breast [11,12], glioma [13,14], and other cancer cells. *N*-methyl-actinodaphine (MA) is an aporphine alkaloid isolated from the roots of *Cinnamomum insularimontanum Hay* [15]. MA possesses cytotoxicity in some cancers, antioxidant activity [15,16], and inhibits bacterial endotoxin [17]. However, the mechanism of action of these compounds against breast cancer is still unclear. In the present study, we investigated whether HCD or MA can enhance TMX activity on ER + and ER − breast cancer cell lines, and its possible molecular mechanism of apoptosis was determined.

## 2. Results and Discussion

### 2.1. HCD, MA, and Tamoxifen Effects on Breast Cancer and Human Mammary Epithelial Cell Viability

**Figure 1:**
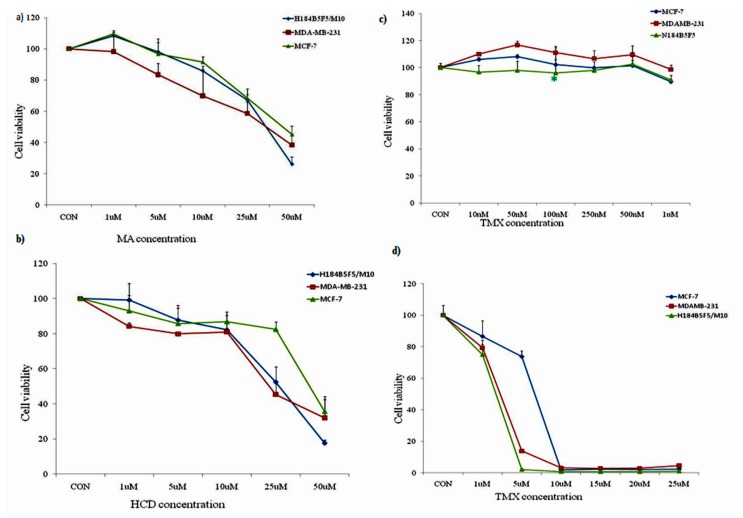
Effect of MA, HCD, and TMX on cell viability of breast cancer cell lines and human mammary epithelial cells by MTT assay. The viability of all the three cell lines was measured after a 24-h treatment with the indicated concentrations of (**a**) MA (1, 5, 10, 25, and 50 µM); (**b**) HCD (1, 5, 10, 25, and 50 µM); (**c**) low dose of Tamoxifen (10, 50, 100, 250, 500, and 1000 nM); and (**d**) high dose of Tamoxifen (1, 5, 10, 15, 25, and 25 μM). Data shown are the mean values ± SE from three independent experiments. Statistical analyses were determined using one-way ANOVA followed by post hoc Tukey multiple comparison test with ** *p* < 0.01, *** *p* < 0.001, significantly different from control.

At first, we examined the potential anticancer effect of MA and HCD in breast ER (+) MCF-7 and ER (−) MDA-MB 231 tumor cells [18] and human mammary epithelial cells by cell viability (MTT) assay. Our results showed that both MA and HCD decreased the viabilities of both types of cancer cells in a concentration-dependent manner (Figure 1a,b). Furthermore, in human mammary epithelial cells, a toxic effect was observed at 50 µM concentration. In all the three cells, the highest dose (50 µM) exhibited the maximum inhibitory effects as compared with the lower doses. Noteworthy, HCD and MA were significantly more active in ER (+) MCF-7 cells at high concentration, the IC_50_ concentration of MA was found to be 25 and 40 μM for MDA-MB 231 and MCF-7 cells, respectively. For HCD, the IC_50_ concentration was found to be 15 and 25 μM for MDA-MB 231 and MCF-7 cells, respectively.

Tamoxifen (TMX) was then used to test the concentration of breast cancer cells (MCF-7 and MDA-MB-231) and human mammary epithelial cells (H184B5F5/M10) in order to facilitate the follow-up experiments. As shown in Figure 1c, the test was carried out at a low concentration of 10 nM, 50 nM, 100 nM, 250 nM, 500 nM, and 1 μM, respectively. It was found that with the increase of TMX concentration; survival rate was not significantly inhibited, indicating that low doses of TMX failed to inhibit the survival rate of breast cancer cells. Therefore, we increased the concentration of TMX (1, 5, 10, 15, 20, and 25 μM) and analyzed cell viability. Increased TMX (>1 μM) concentration showed higher cytotoxic effect to normal mammary epithelial cells and breast cancer cells (Figure 1d). At 24 h, about 15% and 20% cell death were recorded with 1 μM tamoxifen, respectively. In the following experiments we used 1 μM of TMX to study the sensitization effect.

### 2.2. MA or HCD Pretreatment Could Enhance the Growth Inhibition of TMX in MDA-MB 231 Breast Cancer Cells

**Figure 2:**
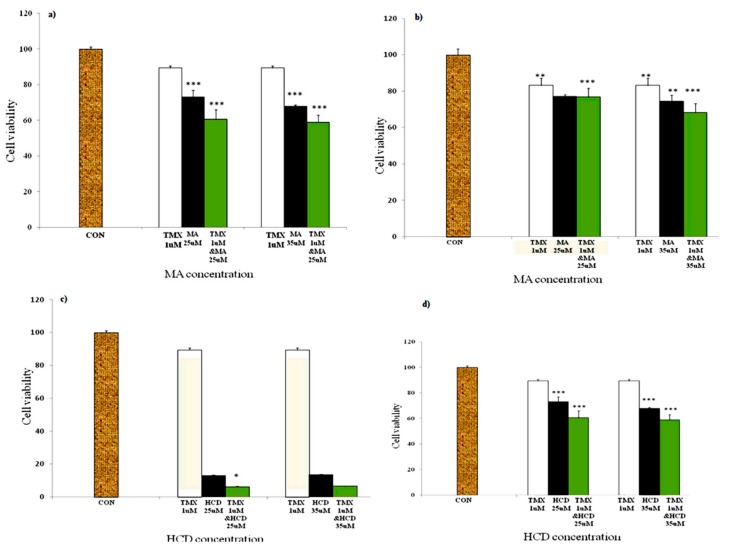
MA/HCD pretreatment increases the efficacy of Tamoxifen. Breast cancer cell lines (**a**) MDA-MB 231 and (**b**) MCF-7 were pretreated with MA for 12 h followed by TMX treatment for additional 12 h. Exposure of breast cancer cells to HCD for 12 h prior to TMX treatment; (**c**) MDA-MB-231 and (**d**) MCF-7 cells as the percentage of viability was reduced when compared to control level. The cell viability was analyzed using MTT assay. *p* values were determined using one-way ANOVA (* *p* < 0.05, ** *p* < 0.01, *** *p* < 0.001 as compared with the control).

To test whether MA or HCD can sensitize MCF-7 and MDA-MB-231 to TMX treatment, MTT assays were performed. Cells were pretreated with increasing concentrations of either MA (25 and 35 μM) or HCD (15 and 25 μM) alone for 12 h and then treated with TMX for 12 h. The results presented in Figure 2a,b showed that MA treatment (*p* < 0.001) enhanced the sensitivity of both breast cancer cell lines to TMX than cells treated with TMX or MA alone. When compared with MA and TMX treatment alone, MA (25 μM) plus TMX (1 μM) inhibited MDA-MB 231 cell growth by ~40% and 20% in MCF-7 cells. Similarly, MA (35 μM) plus TMX (1 μM) treatment inhibited MDA-MB 231 cell growth by ~45% and ~30% in MCF-7 cells after 24 h of treatment.

Next, we tested whether HCD could sensitize breast cancer cell lines to the TMX treatment. Surprisingly, we found that HCD treatment alone caused higher cell death; the cell death was increased in HCD plus TMX treated ER (−) cells (Figure 2c,d). No significant difference in cell death was observed at high HCD concentration. In MCF-7 cells, HCD (35 μM) plus TMX (1 μM) treatment significantly inhibited cell viability than HCD (25 μM) plus TMX (1 μM) treatment. Triple negative breast cancer is one of the most aggressive and complicated types of breast cancer to treat [8]. From our cell viability assay it is clear that both MA and HCD have the ability to sensitize TMX in inhibiting triple negative breast cancer MDA-MB 231 cells than estrogen (+) MCF-7 cells. Furthermore, we also determined the effect of TMX and MA or HCD on normal mammary epithelial cells (data not shown). No significant changes were observed between the control and treatment groups. 

### 2.3. Effect of MA or HCD on Tamoxifen Treated Breast Cancer Cell Cycle Distribution

**Figure 3:**
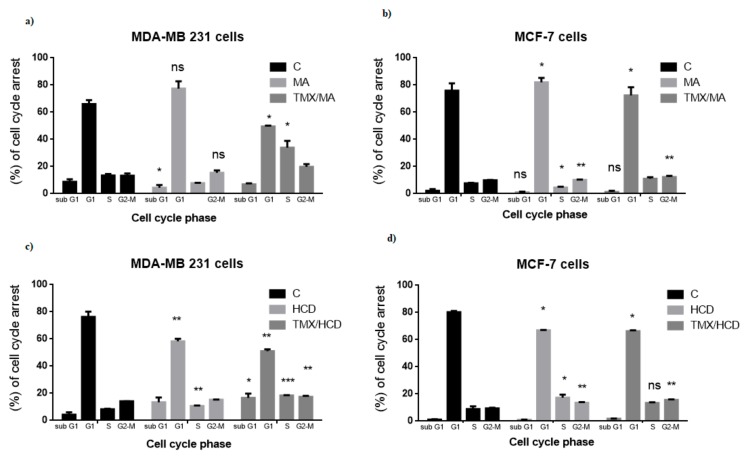
Effect of MA or HCD on TMX treated cell cycle progression (**a**) MDA-MB 231 breast cancer cells and (**b**) MCF-7 cells were treated with MA (25, 40 μM) alone for 24 h or pretreated with MA for 12 h and then treated with TMX for another 12 h and (**c**) MDA-MB-231 breast cancer cells and (**d**) MCF-7 cells were treated with HCD (25 μM) alone and in combination with Tamoxifen (1 μM) for 24 h. Cells are then analyzed for cell cycle arrest by Flow cytometry with propidium iodide staining. Data shown are the mean values of two individual experiments ± SD values which were determined using one-way ANOVA (* *p* < 0.05, ** *p* < 0.01, ** *p* < 0.001 as compared with the control group).

Many studies suggest that cell cycle arrest leads to cell growth inhibition or apoptosis. Thus, cell cycle arrest is a potential marker for chemical or drugs anti-tumor activity [19]. The effects of MA and/or TMX or HCD and/or TMX on MDA-MB 231 and MCF-7 cell cycle were analyzed. MA treated MDA-MB 231 (IC_50_ of MA 25 µM) and MCF-7 (IC_50_ of MA 40 µM) cells had an increased percentage of G_1_ phase compared to untreated control. Combination of MA and TMX (MA 25 µM + TMX 1 µM for MDA-MB 231) had a significantly higher percentage of S and G2-M phase (30.3 & 18.3%) than cells treated with combination of MA 40 µM and TMX 1 µM and drug alone treated cells (Figure 3a,b). The combination index between TMX and MA was 0.97 (MDA-MB 231) and 0.89 (MCF-7), which strongly indicated synergistic enhancement of cytotoxicity in TMX/MA combination.

Similarly, HCD treatment alone increased G1 phase in both MDA-MB 231 and MCF-7 cells (Figure 3c,d). HCD and TMX (HCD 15 µM + TMX 1 µM for MDA-MB 231) combinations increased S and G2-M phase (18.3% & 17.9%) compared to HCD treatment alone. In MCF-7 cells, HCD treatment (IC_50_ of HCD 25 µM) alone increased S and G2-M phase, interestingly HCD plus TMX combination increased G2-M phase (15.3%). Collectively, these results suggest that both MA and HCD enhance TMX by inducing triple negative breast cancer—MDA-MB 231 cells cycle arrest at G2-M phase in comparison to MCF-7 cells.

Then, the combination index between TMX and HCD was calculated and the result was 0.91 in MDA-MB-231 and 0.78 in MCF-7, which also revealed the synergistic effect in TMX/HCD combination. Furthermore, we also determined the effect of TMX and MA or HCD on normal mammary epithelial cells. No significant changes were observed between the control and treatment groups (data not shown).

### 2.4. Effect of MA/HCD and or TMX on Apoptotic Markers

**Figure 4:**
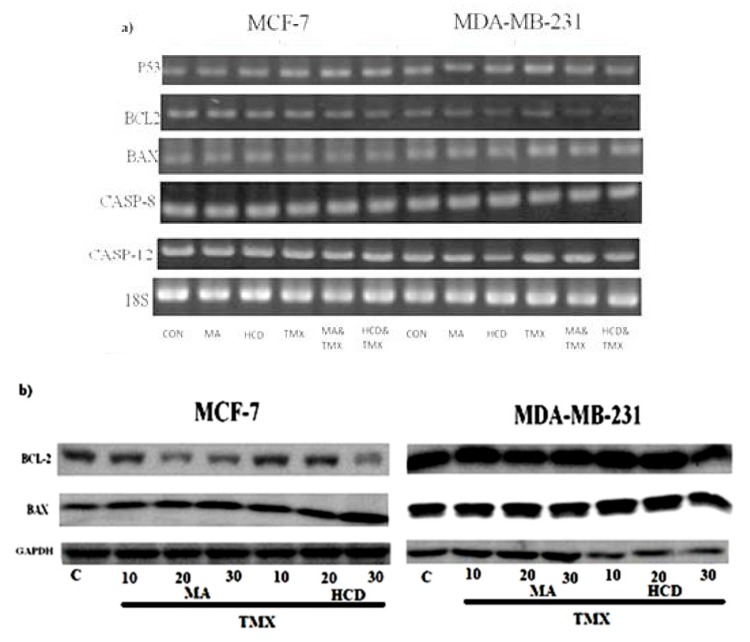
Breast cancer cells were treated with MA or HCD alone or in combination with TMX for 24 h and then analyzed for (**a**) mRNA (p53, Bcl2, Bax, Cas-8, Cas-12); (**b**) MCF-7 and MDA-MB 231 cells were treated with various concentrations of MA and HCD (10, 20, and 30 μM) for 12 h and further treated with TMX (1 μM) for additional 12 h and then analyzed for BAX and BCL-2 protein expression. For mRNA expression 18S was used as an internal control and for Western blotting GAPDH was used as an internal control, respectively. Data shown are the mean ± SD values of three independent experiments (* *p* < 0.05, ** *p* < 0.01 as compared with the untreated control).

Previous studies have shown that p53 has the ability to induce apoptosis by activating various proapoptotic genes such as Bax or by suppressing anti-apoptotic genes of the Bcl-2 family [20,21]. Altered expression of Bcl-2 and Bax may result in resistance to a wide variety of cell death stimuli including chemotherapeutic drugs [22]. Therefore, in the present study, we investigated the expression of the pro-apoptotic Bax and anti-apoptotic Bcl-2 and tumor suppressor p53 genes’ expressions in MA/HCD, TMX and MA/HCD plus TMX treated cells. When compared with HCD or TMX treatment, HCD plus TMX treatment showed significant increase in p53 and Bax mRNA expression and decreased Bcl-2 mRNA expression in breast cancer cells (Figure 4a). A similar result was found in MA plus TMX treated group. Numerous studies have demonstrated that dysregulated caspase activity in breast cancer is involved in chemotherapeutic resistance [23]. Next, we evaluated the effect of these drugs on TMX treated breast cancer cells by analyzing caspases 8 and 12 mRNA expression. MA, HCD, MA plus TMX, and HCD plus TMX did not affect Caspase-8 and Caspase-12 mRNA expression.

We further confirmed the expression of Anti-apoptotic protein- Bcl-2 and pro-apoptotic Bax protein expression by Western blot. As shown in Figure 4b, MA or HCD plus TMX breast cancer cells showed decreased BCL-2 protein expression and increased BAX expression.

## 3. Materials and Methods

### 3.1. Cell Culture

H184B5F5/M10 human mammary epithelial cells, MCF-7 (ER positive), MDA-MB 231 (triple negative including ER) human breast cancer cells were obtained from the American Type Culture Collection (Rockville, MD, USA). This cell line was cultured in RPMI 1640 medium supplemented with 10% heat inactivated fetal bovine serum (FBS), 100 IU/mL penicillin, 2 mM l-glutamine, 10 mM HEPES, and 1.0 mM sodium pyruvate in a 5% CO_2_ incubator at 37 °C.

### 3.2. Cell Viability Assays

Cells were seeded in 96-well plates and grown overnight and then incubated in 10% FBS media containing different amounts of Tamoxifen (TMX), MA, HCD, or TMX/MA, TMX/HCD for 24 h. After incubation, 2 mg/mL of (3-(4,5-dimethylthiazol-2-yl)-2,5-diphenyltetrazolium bromide) MTT reagent was replaced and incubated in a 5% CO_2_ incubator at 37 °C for another 4 h. The cells were harvested in 50 μL/L of DMSO and absorbance was measured at 570 nm by using a microplate reader (Molecular Probes Inc., Eugene, OR, USA). The synergistic effect between TMX, MA, and HCD were measured as IC_50_ of the compounds alone (HCD, MA, TMX) and in combination (TMX/HCD and TMX/HCD). Then, the combination index (CI) was calculated based on the determined IC_50_. CI value specified the drug–drug interaction whereas CI < 1 as synergism, CI = 1 as additive, CI > 1 as antagonism [24].

### 3.3. Analysis of Cell Cycle Distribution

Cells were cultured and then treated with or without Tamoxifen (TMX), HCD or TMX/HCD for 24 h in 10% FBS media. Cells were fixed with 70% ethanol overnight ethanol. After an additional wash with PBS, the cell pellets were stained with 1 mL of propidium iodide (PI) staining solution containing 200 μg of PI in 1 mL of PBS containing 2 mg of DNase free RNase for 30 min. Cell cycle distribution was analyzed by FACS flow cytometry (Becton Dickinson, Mountain View, CA, USA).

### 3.4. RT-PCR

Total RNA was isolated after treatment using the Trizol reagent (Invitrogen, Burlington, ON, USA) according to the manufacturer’s instruction. Isolated RNA was used as a template for the reverse transcription reaction (Invitrogen, Carlsbad, CA). RT was performed at 37 °C for 60 min using 55.5 μL DEPC H_2_O, 4 μg total RNA, 0.5 μL of RNase inhibitor (40 U/μL) (Promega, Madison, WI, USA), 20 μL of 5X RT buffer, 8 μL of dNTP (2.5 mM), 10 μL of oligo(dT) (5 μM/mL) (Mission Biotech, Taipei, Taiwan), and 2 μL of MMLV reverse transcriptase (200 U/μL) (Promega). The resulting cDNA was added to the PCR mixture containing 9.5 μL of DEPC water, 2.5 μL of 10X PCR buffer (MD Bio, Taipei, Taiwan), 2.5 μL of dNTP (10 mM) (Promega), 2.5 μL of each primer (5 μM), 0.5 μL of Taq (2 U/μL) (MD Bio), and 4 μL of 2.5 mMdNTP mixture. After 94 °C, 5 min denaturing, 94 °C, 45 sec denaturing, 55 °C, 1 min annealing, and 72 °C, 1 min extension were repeated 35 cycles and final step was 72 °C 10 min extension. The PCR products were run on 1.5% agarose by electrophoresis and the amplicon was analyzed by gel analysis system for image acquisition (Evergene, Taiwan). After internal control (18 s rRNA) normalization, the intensity of the tested genes was quantitively analyzed by Phoretix gel analysis software 1D standard (version 3.00, Newcastle, UK).

### 3.5. Western Blot Analysis

After treatment, cells were collected and washed twice in 1× PBS, then lysed in ice-cold lysis buffer (50 mM Tris–HCl, pH 7.4, 150 mM NaCl, 5 mM EDTA, 50 mM NaF, 1%Triton X-100, 1 mM sodium orthovanadate, 1 mM phenylmethanesulfonyl fluoride, 1 mg/mL aprotinin, 2 µg/mL pepstatin A, and 2 µg/mL leupeptin) for 5 min. Thirty µg proteins were separated using sodium dodecyl sulfatepolyacrylamide gel electrophoresis (10% SDS-PAGE, 100 V) and subsequently transferred to PVDF (Millipore, Bedford, MA, USA) membrane. The blots were blocked with 5% non-fat milk in TBST saline (20 mM Tris–HCl, pH 7.4, 137 mM NaCl, and 0.05% Tween-20) at RT for 1 h and incubated with the appropriate primary antibody at 4 °C. After washing, the blots were incubated with peroxidase conjugated secondary antibody for 1 h. Bands were monitored using Western blot chemiluminescence reagent (Amersham Biosciences Corp., Piscataway, NJ, USA). After internal control (GAPDH) normalization, the signals were quantitively analyzed by image analysis system (PerkinElmer Life Sciences, Boston, MA, USA).

### 3.6. Statistical Analysis

Data were expressed as the mean ± SD. Statistical analysis was conducted by using one-way analysis of variance (ANOVA) and by Tukey test. A probability level of *p* < 0.05 was considered statistically significant.

## 4. Conclusions

Thrombosis, pancreatitis due to hypertriglyceridemia, increasing risk of Parkinson’s disease, uterine malignancies, and insomnia are considered side effects of tamoxifen treatment [25,26,27,28,29]. Due to ER agonist nature, tamoxifen could reduce ER positive breast cancer growth but about 5–10% of ER negative breast cancer is sensitive to tamoxifen [30]. Moreover, tamoxifen-resistant breast cancer could be induced after long-term administration, which is a severe issue for breast cancer treatment [31]. Thus, these experiments attempted to determine whether the combination of MA or HCD with TMX can sensitize ER positive (MCF-7) and ER negative (MDA-MB231) cells to the chemotherapy drug TMX. This study provides experimental support showing that 16-hydroxycleroda-3,13-dien-15,16-olide (HCD) or *N*-methyl-actinodaphine (MA) can be used to sensitize aggressive breast cancer (MDA-MDB-231) cells to tamoxifen treatment, which may represent an alternative approach to destroy triple negative breast tumors in patients with chemotherapy resistance. Inhibitory effects of MA or HCD on breast cancer cell lines, specifically the ERα-negative MDA-MB-231 and ERα-positive MCF-7 and the molecular targets and signaling pathways include anti-apoptotic genes-Bcl-2, pro-apoptotic-Bax, and tumor suppressors (such as p53), were investigated. Most importantly, human triple negative breast cancer cells were more efficiently destroyed than ER (+) breast cancer cells by the combined therapy of MA or HCD with TMX. Further studies are required to elucidate how the combination of HCD, MA, and tamoxifen can regulate various molecular mechanisms.
